# Zebrafish Model Reveals Early Electrocardiographic and Molecular Signatures of Doxorubicin-Induced Cardiotoxicity

**DOI:** 10.1007/s12012-026-10113-y

**Published:** 2026-04-13

**Authors:** Zih-Yin Lai, Chi-Ying Lee, Yu-Ching Chiu, Chia-Hung Lin, Chung-Chi Yang, Chia-Chia Ho, Lawrence Yu-Min Liu, Yung-Jen Chuang

**Affiliations:** 1 School of Medicine, National Tsing Hua University, Hsinchu, 300044 Taiwan, R.O.C.; 2 Institute of Bioinformatics and Structural Biology, National Tsing Hua University, Hsinchu, 300044 Taiwan, R.O.C.; 3 Division of Cardiovascular Medicine, Taoyuan Armed Forces General Hospital, Taoyuan City, 325208 Taiwan, R.O.C.; 4 Cardiovascular Division, Tri-Service General Hospital, National Defense Medical Center, Taipei City, 114201 Taiwan, R.O.C.; 5 Division of Cardiology, Department of Internal Medicine, Hsinchu MacKay Memorial Hospital, Hsinchu, 300044 Taiwan, R.O.C.; 6 Department of Medicine, MacKay Medical College, New Taipei City, 252005 Taiwan, R.O.C.

**Keywords:** Doxorubicin, Cardiotoxicity, Zebrafish, Electrocardiography, PR interval

## Abstract

Doxorubicin (DOX) is a highly effective anthracycline widely used in cancer therapy but limited by its dose-dependent cardiotoxicity, which may result in arrhythmia, dilated cardiomyopathy, and heart failure. Conventional surveillance tools, including echocardiography and serum biomarkers, often identify injury only after substantial cardiac dysfunction has occurred. This underscores the need for early markers with mechanistic relevance. In this study, we developed an integrated zebrafish platform combining pathophysiological evaluation, electrocardiography (ECG), and transcriptomic profiling to establish a novel approach for early detection of DOX-induced cardiotoxicity (DIC). Consistent with human and mammalian models, DOX administration in adult zebrafish resulted in ventricular enlargement, myocardial fiber disarray, and elevated troponin I levels. ECG recordings revealed dose-dependent conduction disturbances, notably progressive PR interval and QRS prolongation, with P wave widening at higher doses. These findings identify the PR interval as a sensitive, early index of conduction impairment in the zebrafish DIC model, consistent with clinical reports linking PR prolongation to adverse outcomes. RNA sequencing further identified transcriptional pathways associated with conduction delay, with dysregulation of sodium channels (*scn5lab*, *scn1lab*), gap junction proteins (*cx43*, *cx40.8*), and transcriptional regulators (*nkx2.5*, *tbx* family). Notably, *scn1lab* expression declined progressively, *cx43* and *nkx2.5* were upregulated, showing temporal changes that co-occurred with the observed ECG and structural phenotypes. Together, these results support adult zebrafish as a scalable platform for cardiotoxicity screening and highlight PR interval prolongation as an early electrophysiological marker of DOX-associated conduction disturbance. The transcriptomic signatures are presented as correlative, hypothesis-generating candidates relevant to cardiac conduction and remodeling.

**Graphical Abstract:**

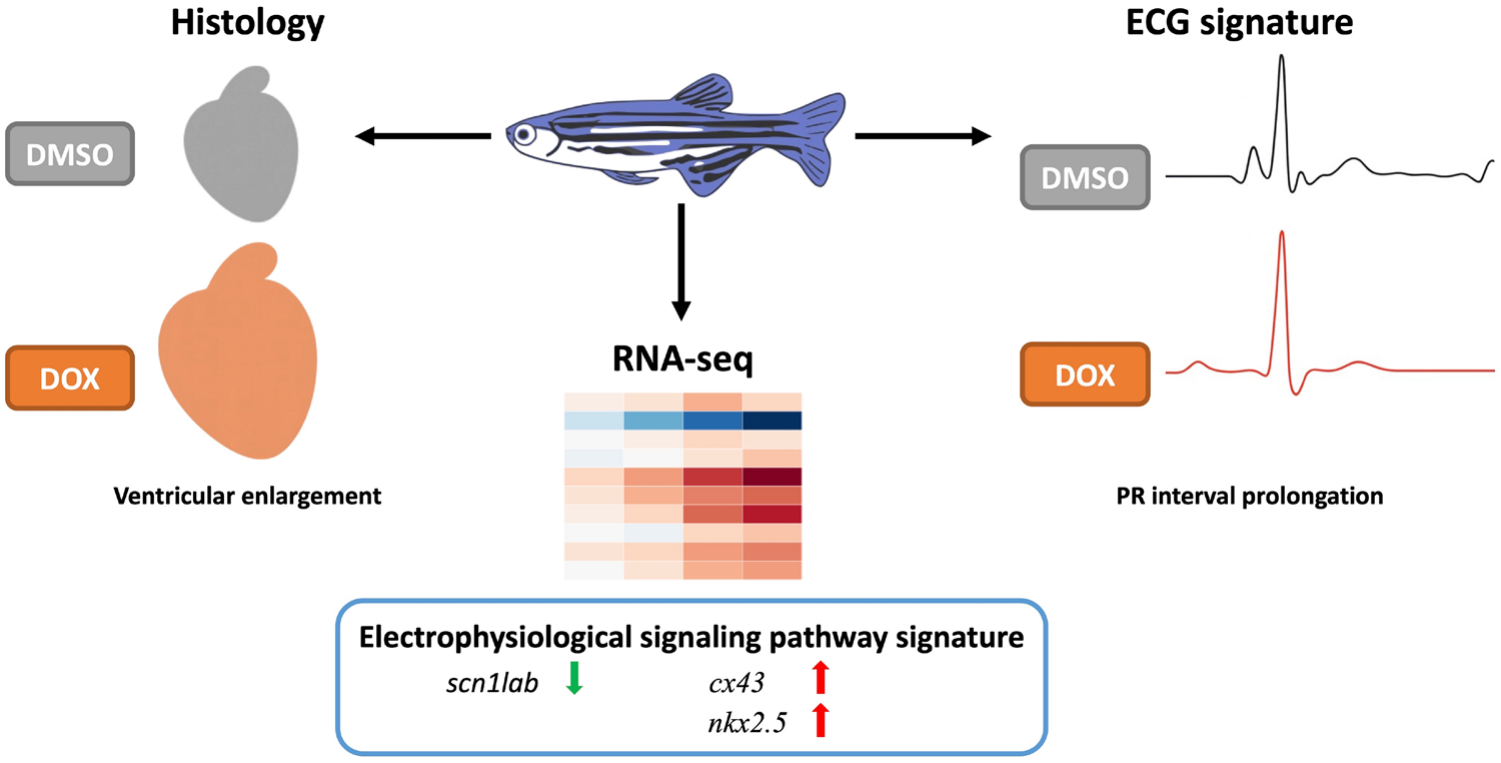

## Introduction

Doxorubicin (DOX) is a highly effective anthracycline chemotherapy agent that is widely used to treat various malignancies, including breast cancer, leukemia, and lymphoma. Despite its clinical efficacy, DOX treatment is significantly limited by its cardiotoxic side effects, which can manifest as arrhythmia, dilated cardiomyopathy, and congestive heart failure. DOX-induced cardiotoxicity (DIC) affects approximately 5–48% of patients receiving anthracycline therapy, with subclinical cardiotoxicity developing in up to 57% of patients of treatment [1, 2]. Given these substantial cardiac risks, early detection and effective monitoring of DIC are essential for optimizing patient outcomes and developing cardioprotective interventions.

Current clinical practice guidelines recommend baseline and periodic echocardiographic monitoring, with treatment modification when left ventricular ejection fraction (LVEF) drops below 50% [3]. However, this approach has significant limitations: echocardiographic changes typically occur only after 20–30% of myocardial function is lost, cardiac biomarkers lack specificity for early detection, and there are no validated predictive models for individual patient risk stratification [4, 5]. The European Society of Cardiology has therefore recommended that all patients undergo electrocardiography (ECG) testing both before and during cancer treatment [6]. Clinical evidence supporting this approach is accumulating; for example, a retrospective study of approximately 600 pediatric cancer patients treated with anthracyclines reported ECG abnormalities, including decreased QRS amplitudes and prolonged QTc (corrected QT) intervals [7]. Clinical and animal studies have further demonstrated that DOX can induce various arrhythmias, including ST interval prolongation, atrioventricular (AV) block, QT prolongation, T wave flattening, and pathological Q waves [8–10].

Although considerable research has investigated the DIC [11, 12], critical knowledge gaps remain unaddressed. Existing preclinical models demonstrate limited correlation between early molecular events and subsequent electrophysiological dysfunction, with most studies focusing on either endpoint in isolation rather than their temporal relationship. Furthermore, the molecular basis of DOX-induced electrophysiological changes, particularly the sequence of molecular events preceding detectable cardiac dysfunction, remains inadequately understood, hindering the development of timely interventions [13]. Additionally, existing animal models using rodents require large sample sizes and extended observation periods, limiting their utility for rapid screening of cardioprotective interventions. These limitations underscore the necessity for improved models and novel biomarkers that can predict the onset and progression of DIC before clinical manifestations arise.

Zebrafish (*Danio rerio*) have emerged as a powerful animal model for cardiovascular research due to their genetic homology to humans, conserved cardiac physiology, and suitability for efficient high-throughput drug screening [14, 15]. Their two-chambered heart exhibits electrophysiological properties similar to those of humans, including a comparable heart rate (120–130 bpm) and ECG waveform patterns that reflect human cardiac activity. Notably, adult zebrafish’s QTc interval, measuring 389 ± 38 ms [16], aligns with human values, indicating similar cardiac depolarization and repolarization processes. In contrast to rodent models, which are increasingly constrained by ethical and economic considerations, zebrafish ECG parameters are more similar to those of humans and enable more efficient screening [17]. Numerous studies have demonstrated that the zebrafish disease model effectively replicates several abnormal ECG signals observed in patients with human cardiovascular disorders [16, 18]. However, previous zebrafish studies have investigated DIC through either histopathological or molecular approaches, without systematically integrating electrophysiological monitoring with temporal molecular profiling to establish predictive relationships.

In this study, we developed an integrated approach combining conventional pathophysiological analysis with molecular profiling and electrophysiological assessment to comprehensively investigate DIC in zebrafish. This integrated methodology allows us to establish causative links between molecular events and functional cardiac changes in a time-resolved manner, providing a mechanistic foundation for early intervention strategies. We first evaluated myocardial damage and well-established cardiac biomarkers associated with DIC in adult zebrafish, then monitored ECG signals at varying DOX concentrations to determine electrophysiological alterations correlated with cardiac injury responses. Simultaneously, we performed gene expression analysis focusing on genes implicated in transient ECG alterations that subsequently normalize, as well as those potentially contributing to the maintenance of redundant or reserve conduction capacity, to delineate the precise sequence of events from molecular initiation to functional manifestation. Our objective is to identify predictive ECG indices and novel biomarkers that can facilitate the early detection of cardiotoxic effects, establish predictive models for early cardiotoxicity detection, and provide translational insights relevant to human cardiac health. We aim to advance the zebrafish model as a reliable and consistent assay system, thereby enabling the accelerated translation of preclinical research findings into clinical applications.

## Materials and methods

### Zebrafish and Ethics Statement

Adult wild-type AB strain zebrafish (6 to 10 months) weighing between 200 and 350 mg were used in this study. Male and female zebrafish were randomly selected for each experiment. All experimental protocols and animal use were approved and followed the guidelines and regulations of the Institutional Animal Care and Use Committee of National Tsing Hua University, Hsinchu, Taiwan, R.O.C. (Approval No. 109023).

### Zebrafish Anesthesia

Anesthesia was administered as previously described [19]. Zebrafish were immersed in a solution containing 70 ppm MS-222 (Sigma-Aldrich, St. Louis, MO, USA) and 70 ppm isoflurane (Baxter, Guayama, PR, USA) for 2–5 min. The level of anesthesia was assessed by applying pressure to the tail of the zebrafish.

### Drug

DOX hydrochloride (Selleck Chemicals, Houston, TX, USA) was dissolved in 2% dimethyl sulfoxide (DMSO) (Sigma-Aldrich, St. Louis, MO, USA) to prepare 10 µg/µL stock solution, which was stored at − 20 °C till use.

### Zebrafish Intraperitoneal (IP) Injection

Intraperitoneal (IP) injection was conducted according to the method outlined in a published protocol [20]. Prior to the procedure, the zebrafish were sedated and secured on a moist sponge with their dorsal side facing upwards. The syringe (26s gauge, Hamilton 701 N; Reno, NV, USA) was washed 3–4 times with 75% ethanol and phosphate-buffered saline (PBS) before each injection. Subsequently, a 4 µL DOX solution was extracted, and the tip of the needle was positioned at the center of the pelvic fins, with the beveled edge facing upward. The needle was inserted 1–2 mm into the abdominal cavity of the zebrafish before administering the solution.

### Zebrafish ECG Recording

The ECG procedure was conducted as described in a previous study [21]. Following anaesthetization of the zebrafish, as previously described, the fish were positioned ventral side up in a Y-shaped groove within a damp sponge. During real-time ECG recording, two needle electrode probes were carefully inserted into the body of the zebrafish, with one placed in the pectoral region and the other in the abdomen. A third needle electrode, which served as the grounding reference, was inserted into the sponge. After the recording was completed, the zebrafish were promptly transferred to a clean recovery tank.

### Cryosection and Hematoxylin and Eosin (H&E) Staining

The heart tissue cryosection protocol was followed as previously described [22]. The hearts were sliced into 12 μm thick sections using a cryostat (CM3050S; Leica Biosystems, Deer Park, IL, USA), and the slides were stored at − 80 °C before staining.

Before H&E staining, the heart sections were dehydrated in a 60 °C oven for 5 min. Through H&E staining, the sections were first incubated with 0.1% Mayer’s hematoxylin (Sigma-Aldrich, St. Louis, MO, USA) for 10 min, covered with foil. After that, the reverse side of the sections was rinsed in running water for 5 min and dipped in 0.5% eosin Y (Sigma-Aldrich, St. Louis, MO, USA) for 12 times. Next, excess eosin was removed by dipping the sections in distilled water. Finally, the sections were dehydrated by dipping them in increasing concentrations of ethanol and washed with xylene. All slides were covered with cover slips using mounting reagent (Fisher Scientific, Fair Lawn, NJ, USA) before imaging using a standard upright microscope (LSM 800; ZEISS, Oberkochen, BW, Germany).

### Zebrafish Cardiac Troponin I Analysis

Adult zebrafish blood was collected using a previously described method with some modifications [23]. Two centrifuge tubes (0.5 mL and 1.5 mL) were prepared for each fish, with the smaller tube perforated and placed inside the larger one. Both tubes were coated with 500 i.u. mL^− 1^ heparin for at least an hour before use. The fish were anesthetized, their tails amputated, and blood was drawn using a 10 µL pipette tip preloaded with 5 µL heparin. The blood was transferred into a 1.5 mL tube, and the amputated fish was placed in a smaller tube, allowing blood to drip into the larger one. The tubes were centrifuged at 100 rpm (15 °C, 5 min), then pooled and centrifuged at 2,000 rpm (4 °C, 10 min). After centrifugation, the transparent light-yellow plasma was collected and stored at − 80 °C for quantitative ELISA analysis.

The Fish High Sensitivity Cardiac Troponin I ELISA kit (MBS1603640; MyBioSource, San Diego, CA, USA) was used to quantify plasma cardiac troponin I levels in zebrafish following the manufacturer’s protocol. Then, the OD value was measured by microplate reader at 450 nm.

### RNA Extraction, Library Preparation, and Sequencing

Zebrafish ventricles from DMSO- and DOX-treated (20 mg/kg) groups were collected at 1, 7, 28, and 56 days post injection (dpi). For both DMSO- and DOX-treated groups, 10–15 ventricles were collected for each time point. Total RNA was extracted using RNAzol^®^ RT (Molecular Research Center, Inc, Cincinnati, OH, USA) according to the manufacturer’s protocol. RNA integrity and concentration were first evaluated from 1 µL aliquots prepared for library construction with the Ovation SoLo RNA-Seq Library Preparation Kit (NuGEN Technologies, Redwood City, CA, USA). The quantity of the resulting libraries was measured using a Qubit 2.0 Fluorometer (Thermo Fisher Scientific, Waltham, MA, USA) in combination with the dsDNA High Sensitivity Assay Kit, while library quality was assessed with an Agilent 2100 Bioanalyzer (Agilent Technologies, Santa Clara, CA, USA) using the DNA 1000 assay. Only libraries with an RNA integrity number (RIN) greater than 7 were advanced to sequencing. For RNA sequencing, biological replicates were defined at the sample level. At each time point, ventricles from 10 to 15 adult zebrafish within the same treatment group were pooled to generate one RNA sample. Pooling was used to reduce inter-individual variability and ensure sufficient RNA yield for sequencing. Each pooled sample was treated as an independent biological replicate for downstream differential expression analysis.

Normalized RNA libraries were clustered and sequenced on an Illumina NovaSeq platform (Illumina, San Diego, CA, USA) using paired-end reads (2 × 150 bp). Each sample generated a minimum of four million high-quality reads. Raw sequencing reads were trimmed with *Trimmomatic* [24], aligned to the zebrafish reference genome (GRCz11) using *HISAT2* [25, 26], and quantified with *featureCounts* (v2.0) [27].

### Differential Expression Analysis

Gene expression data were normalized using the Relative Log Expression (RLE) method implemented in the DESeq2 framework [28, 29]. Log2-transformed expression ratios between DOX- and DMSO-treated groups were calculated at each time point. Genes were classified as differentially expressed if their log2 fold change (Log2 FC) exceeded two standard deviations (SD) from the mean distribution of expression ratios.

### Definition of PR Interval-Associated Gene Set

PR interval-associated genes were curated a priori based on published evidence linking these genes to AV conduction/PR interval regulation, including ion-channel genes involved in atrial depolarization and AV conduction (e.g., sodium and calcium channels), gap-junction genes mediating intercellular electrical coupling, and transcriptional regulators implicated in cardiac conduction system development and maintenance. Candidate genes were compiled from prior PR interval/AV conduction studies and conduction-pathway annotations, and zebrafish orthologs were mapped accordingly [30–32].

### Statistical Analysis

Figures and statistical analyses were generated using GraphPad Prism version 9 (GraphPad, San Diego, CA, USA), and the data were presented as mean ± SD. Multiple *t*-test were used to compare the differences between the two groups. *p* < 0.05 was considered statistically significant.

## Results

### DOX Induced Myocardial Changes in Adult Zebrafish

DIC in adult zebrafish is established by morphological and histological analyses [33]. To validate myocardial damage induced by our experimental protocol, we administered a single injection of 20 mg/kg DOX, as in previous studies [34, 35], and observed cardiac alterations consistent with those reported in zebrafish, humans, and other animal models.

**Fig. 1:**
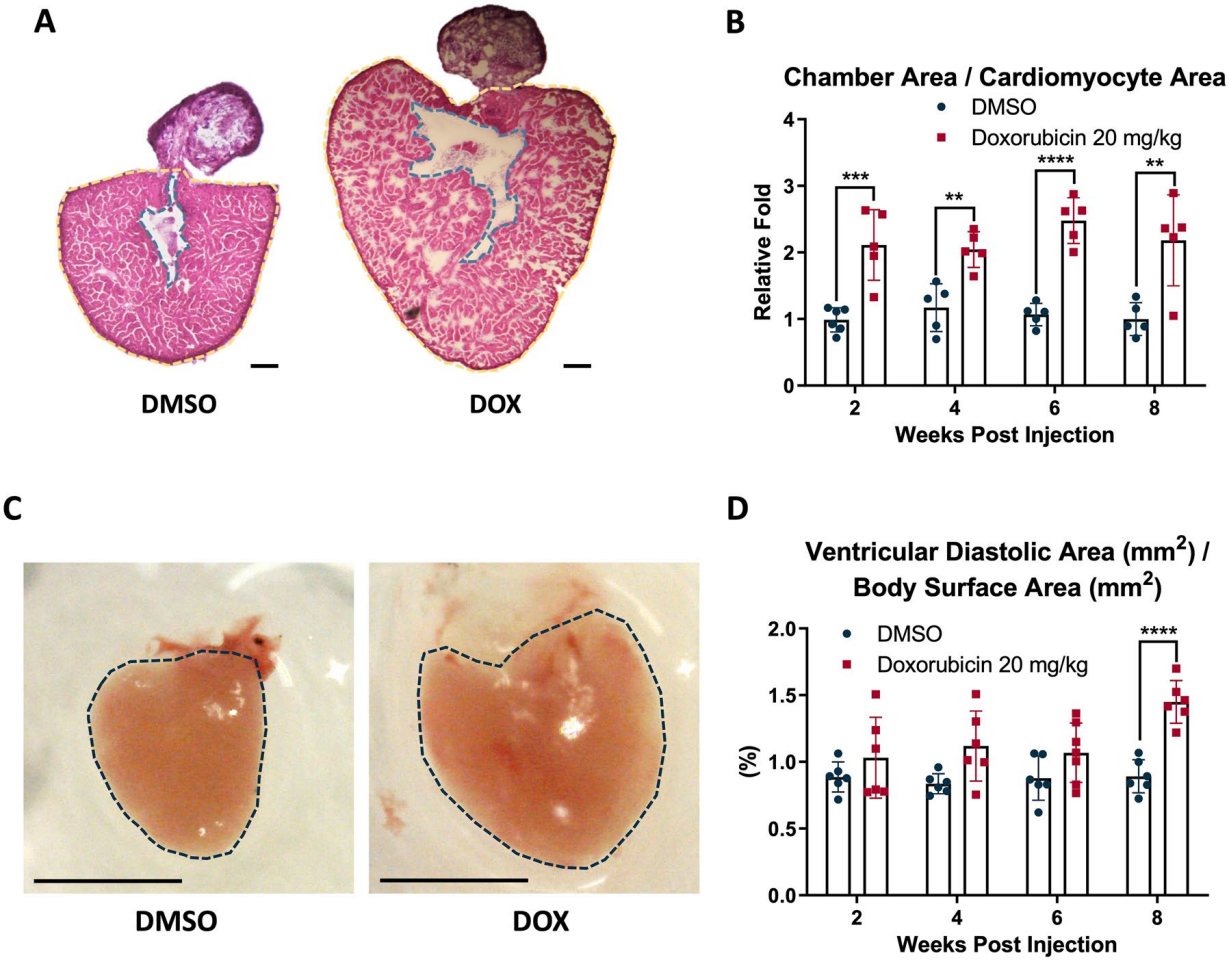
Quantification of ventricle area and chamber-to-cardiomyocyte area ratio in adult zebrafish hearts after 2% DMSO or DOX (20 mg/kg) exposure. **A** The heart sections were stained with H&E. The chamber was demarcated by the dashed blue line, and the cardiomyocyte area was defined as the remaining region within the dashed yellow line, excluding the chamber area. Scale bar: 100 μm. **B** Quantitative analysis of the chamber-to-cardiomyocyte area ratio at different time points after DOX treatment. At 2, 4, 6, and 8 wpi, the fold changes of the chamber area relative to the cardiomyocyte area were 2.11 ± 0.53, 2.04 ± 0.27, 2.48 ± 0.34, and 2.18 ± 0.68, respectively. **C** Representative images of dissected zebrafish hearts with ventricle boundaries delineated by dashed blue lines at 8 weeks after 2% DMSO or DOX 20 mg/kg treatment. Scale bar: 1 mm. **D** The quantitative analysis of ventricle area, comparing DMSO- and DOX-treated groups. At 8 wpi, the ratio of ventricular diastolic area to body surface area was significantly higher in the DOX group (1.45 ± 0.16%) compared to DMSO (0.89 ± 0.12%). (**p < 0.01 vs. DMSO; ***p < 0.001 vs. DMSO; ****p < 0.0001 vs. DMSO; *n* = 3 per group)

Dilated cardiomyopathy, frequently observed in DOX-treated patients, manifests as ventricular chamber enlargement and wall thinning due to cardiomyocyte stretching and a reduction in contractile myofibrils [36]. Previous animal studies have confirmed that increased chamber size is an indicator of dilated cardiomyopathy [37, 38]. To investigate whether DOX induces similar effects, we assessed heart chamber areas normalized by cardiomyocyte areas. At 2, 4, 6, and 8 weeks post injection (wpi) of DOX 20 mg/kg, significantly increased by 2.11-, 2.04-, 2.48-, and 2.18-fold, respectively, were observed in the DOX-treated zebrafish compared to the DMSO group (Fig. 1A, B), suggesting DIC consistent with dilated cardiomyopathy.

DOX-induced dilated cardiomyopathy often accompanies ventricular hypertrophy [39]. Previous research demonstrated ventricle enlargement in zebrafish following DOX exposure [34]. Since zebrafish heart size relates proportionally to body size, ventricular area at diastole was normalized to body surface area. At 8 wpi, the relative ventricular size significantly increased in the DOX group (1.45 ± 0.16%) compared to DMSO group (0.89 ± 0.12%), though earlier time points (2, 4, and 6 wpi) showed no significant differences (Fig. 1C, D).

**Fig. 2:**
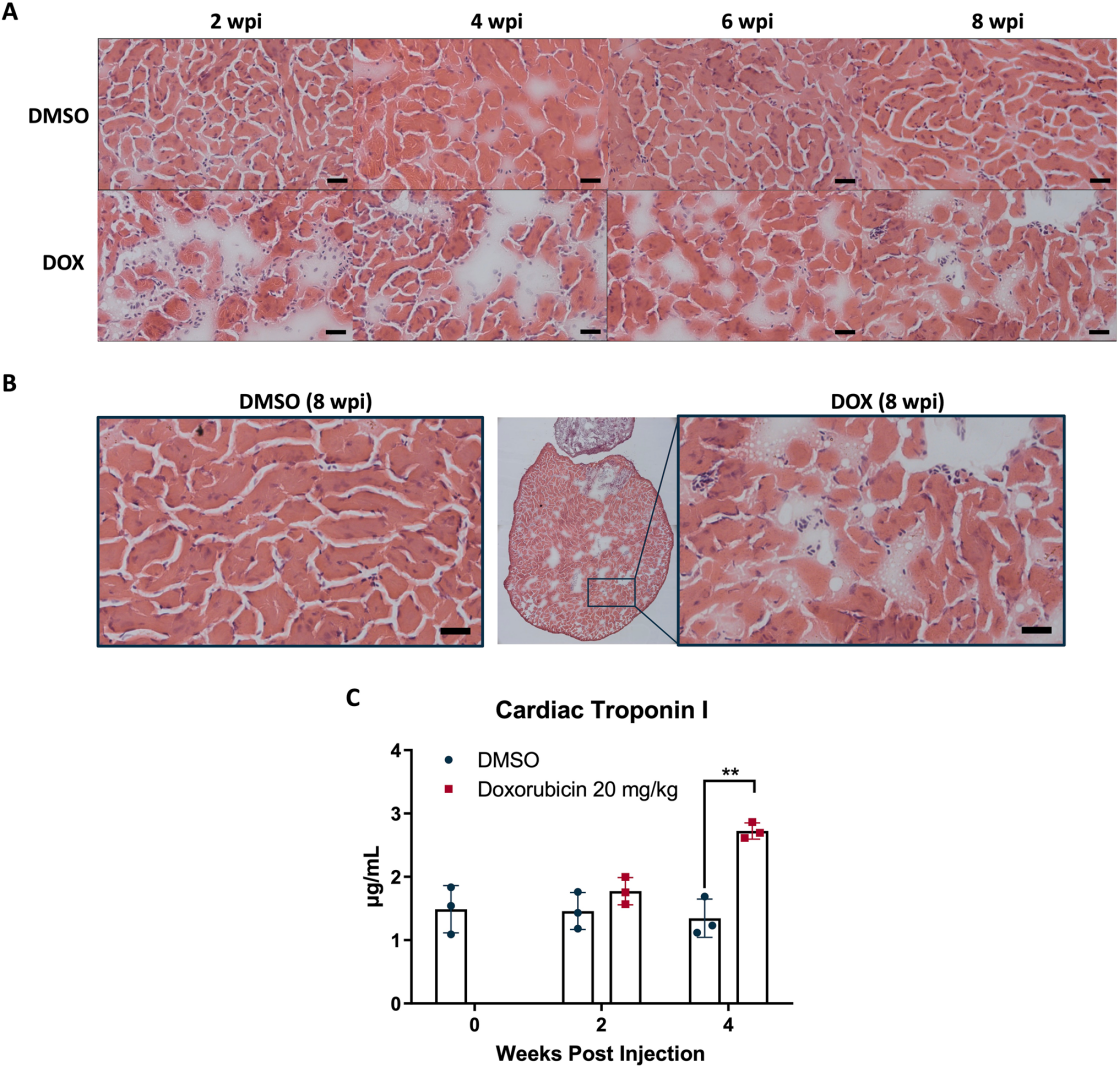
Histological changes and myocardial injury markers following DOX treatment in adult zebrafish hearts. **A** Representative H&E staining of ventricular tissue at 2, 4, 6, and 8 wpi of either 2% DMSO or DOX (20 mg/kg). DOX-treated hearts exhibited enlarged interstitial spaces and disrupted myocardial architecture compared with DMSO groups. Scale bar: 20 μm. **B** High-magnification views at 8 wpi showing preserved architecture in DMSO-treated hearts, whereas DOX-treated hearts displayed structural disorganization and tissue rarefaction. Scale bar: 20 μm. **C** Quantification of plasma cardiac troponin I levels at baseline, 2 wpi, and 4 wpi. Four weeks after DOX (20 mg/kg) treatment, troponin I concentration dramatically increased (2.72 ± 0.13 µg/mL) compared with DMSO (1.38 ± 0.27 µg/mL). (**p < 0.01 vs. DMSO; *n* = 4–12 per group)

Several studies have shown that DOX induces cytoplasmic vacuolization and myofibrillar loss under histological examination [40, 41]. Our findings revealed similar cardiac lesions. In DOX 20 mg/kg-treated hearts, ventricular cardiomyocyte density was significantly lower than DMSO at 2, 4, 6, and 8 wpi, though cell size and inter-bundle gaps remained unchanged (Fig. 2A). This reduction implied myofibrillar loss. Additionally, myocardial vacuolization was observed as early as 2 wpi and became more pronounced by 8 wpi, with numerous vacuoles evident in cardiomyocyte tissue (Fig. 2B; Table 1).

**Table 1:** The frequency of detecting myocardial damage after 20 mg/kg DOX treatment

Frequency of detecting myofibrillar loss and myocardial vacuolization (*n*/*N*, %)				
Time	2 wpi	4 wpi	6 wpi	8 wpi
Myofibrillar loss	5/6 (83%)	6/6 (100%)	6/6 (100%)	4/4 (100%)
Myocardial vacuolization	6/6 (100%)	5/6 (83%)	2/6 (33%)	4/4 (100%)

*n* = number of zebrafish with myocardial damage, *N* = number of observed zebrafish

These findings suggest that DOX induces progressive cardiotoxicity in adult zebrafish with early injury responses and subsequent remodeling features over the 8-week observation window, resembling aspects of dilated cardiomyopathy. The observed structural changes, including ventricular enlargement, myocyte loss, and vacuolization, are consistent with remodeling that may contribute to longer-term functional impairment, although clinical cumulative-dose chronic cardiomyopathy is not fully modeled by this single-dose regimen.

### DOX Induced Cardiac Troponin I Elevation in Adult Zebrafish

Cardiac troponins are among the most sensitive and commonly utilized biomarkers for detecting myocardial injury in clinical settings. Elevated cardiac troponin levels can be observed in various cardiac disorders, including myocardial infarction, heart failure, pericarditis, and tachyarrhythmias [42]. Compared to troponin T, troponin I is regarded as more specific for identifying cardiovascular diseases [43]. Several studies have demonstrated that DOX treatment can lead to increased cardiac troponin I levels in both humans and animal models [44, 45]. Accordingly, we assessed plasma cardiac troponin I concentrations to evaluate DOX-induced cardiac injury in zebrafish. At 2 wpi, the mean troponin I value in the 20 mg/kg DOX-treated group was 1.77 ± 0.22 µg/mL, slightly higher than the DMSO group (1.46 ± 0.29 µg/mL). By 4 weeks after treatment, plasma troponin I levels had risen to 2.72 ± 0.13 µg/mL, which was significantly higher than the DMSO group (Fig. 2C).

### DOX Induced Dose-Dependent ECG Alterations

Among heart screening tests, ECG is the fastest and simplest method to monitor cardiac function and detect damage [46]. Understanding ECG indices linked to cardiac injury enables physicians to identify abnormal signals and adjust treatment before irreversible harm occurs. Therefore, we aimed to investigate ECG alterations after validating our zebrafish model of DIC. Since DIC is known to be dose-dependent, we aimed to determine whether the severity or frequency of abnormal ECG signals varies between low and high DOX concentrations. Therefore, alongside the 20 mg/kg dose, we also assessed ECG markers at a higher DOX dose (40 mg/kg) in adult zebrafish.

**Fig. 3:**
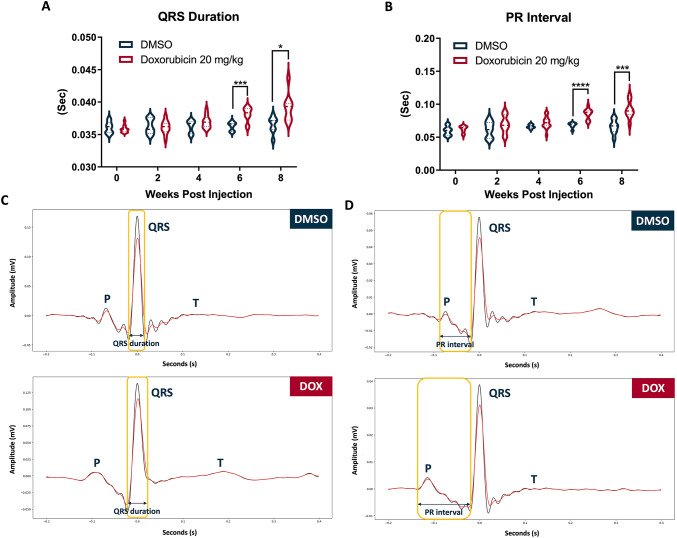
Prolongation of QRS duration and PR interval in 20 mg/kg DOX-treated adult zebrafish. **A** Temporal changes in QRS duration up to 8 wpi of 2% DMSO or DOX (20 mg/kg). DOX treatment significantly increased QRS duration compared with DMSO at 6 and 8 wpi. **B** Temporal changes in PR interval following DMSO or DOX exposure. DOX-treated groups exhibited a progressive prolongation of the PR interval, reaching statistical significance from 6 wpi onward. **C** Representative ECG waveforms showing QRS duration in DMSO- and DOX-treated zebrafish. **D** Representative ECG waveforms illustrating PR interval in DMSO- and DOX-treated zebrafish. (*p < 0.05 vs. DMSO; ***p < 0.001 vs. DMSO; ****p < 0.0001 vs. DMSO; *n* = 5–6 for DMSO group; *n* = 9–19 for DOX group)

Among the key ECG parameters measured after treatment with 20 mg/kg DOX, both QRS duration and PR interval progressively increased over time, becoming significantly prolonged by 6 wpi. At 6 wpi, the mean QRS duration in the DOX-treated group reached 38.11 ± 1.13 millisecond (ms), significantly longer than the DMSO (36.26 ± 0.65 ms), and further increased to 39.21 ± 2.16 ms at 8 wpi (Fig. 3A, C). Similarly, the PR interval was markedly prolonged, averaging 85.3 ± 7.95 ms at 6 wpi versus 68.07 ± 4.04 ms in DMSO, and rising to 91.4 ± 13.56 ms at 8 wpi (Fig. 3B, D).

**Fig. 4:**
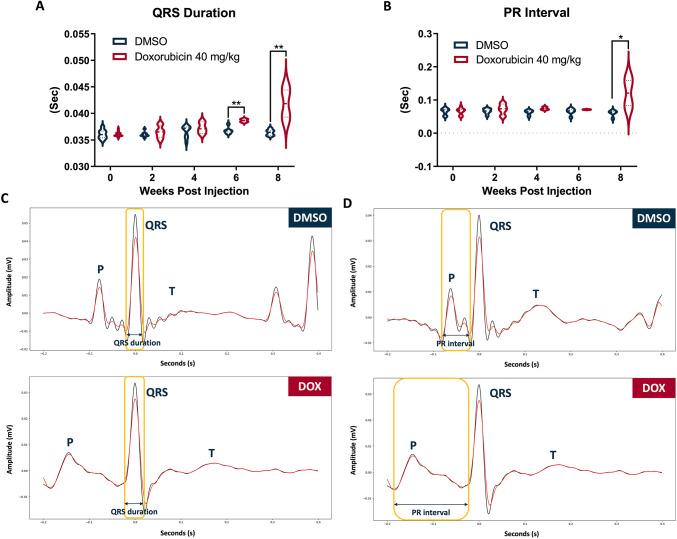
High-dose DOX (40 mg/kg) exacerbated conduction abnormalities in adult zebrafish. **A** QRS duration progressively increased following DOX (40 mg/kg) injection compared with 2% DMSO, with significant prolongation observed at 6 and 8 wpi. **B** PR interval also displayed a marked elevation at 8 wpi in the DOX group, indicating impaired AV conduction. **C**, **D** Representative ECG traces highlighting QRS duration (**C**) and PR interval (**D**) differences between DMSO- and DOX-treated zebrafish. (*p < 0.05 vs. DMSO; **p < 0.01 vs. DMSO; *n* = 7 for DMSO group; *n* = 19–21 for DOX group)

**Fig. 5:**
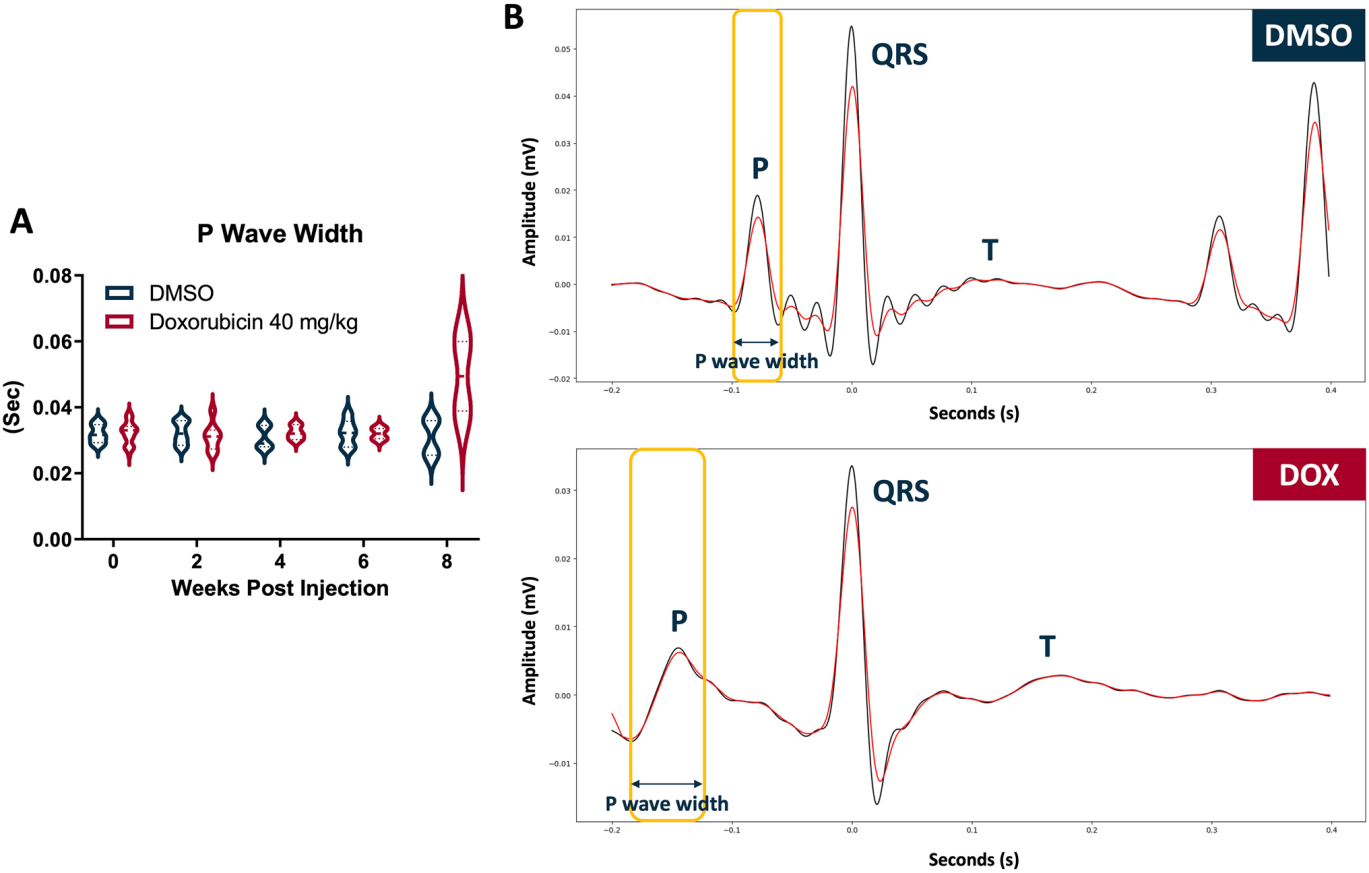
Prolongation of P wave width in adult zebrafish following high-dose DOX. **A** Time course of P wave width changes after injection of 2% DMSO or DOX (40 mg/kg). A slight increase in P wave width was observed at 8 wpi in DOX-treated fish, suggesting impaired atrial conduction. **B** Representative ECG waveforms from DMSO- and DOX-treated zebrafish illustrating differences in P wave morphology and width. (*n* = 7 for DMSO group; *n* = 19–21 for DOX group)

On the other hand, similar to the findings with the lower DOX concentration, QRS duration and PR interval progressively increased following treatment with 40 mg/kg DOX. At 6 wpi, the mean QRS duration in the 40 mg/kg group was 38.65 ± 0.35 ms, significantly longer than the DMSO (36.76 ± 0.64 ms). By 8 wpi, it rose markedly to 41.85 ± 3.61 ms (Fig. 4A, C), exceeding both the DMSO and the 20 mg/kg group (39.21 ± 2.16 ms). Although the PR interval did not differ significantly from DMSO at 6 wpi, it increased substantially by 8 wpi to 120.95 ± 53.8 ms (Fig. 4B, D), significantly higher than the 91.4 ± 13.56 ms observed after 20 mg/kg DOX treatment. In addition to changes in QRS duration and PR interval, the P wave also widened by 8 wpi following 40 mg/kg DOX treatment. At 8 wpi, the mean P wave width in the DOX group was 49.45 ± 14.95 ms, exceeding the DMSO value of 29.12 ± 6.55 ms (Fig. 5). However, the data analysis showed no statistically significant differences between the groups at any time point.

These data suggest that DOX causes dose-dependent, progressive conduction abnormalities in zebrafish, shown by prolonged QRS duration and PR interval. Higher doses led to more marked ECG changes. Overall, ECG parameters are sensitive markers of DIC.

### PR Interval and Heart Rate as Sensitive Indicators of DOX-Induced Conduction Abnormalities

**Fig. 6:**
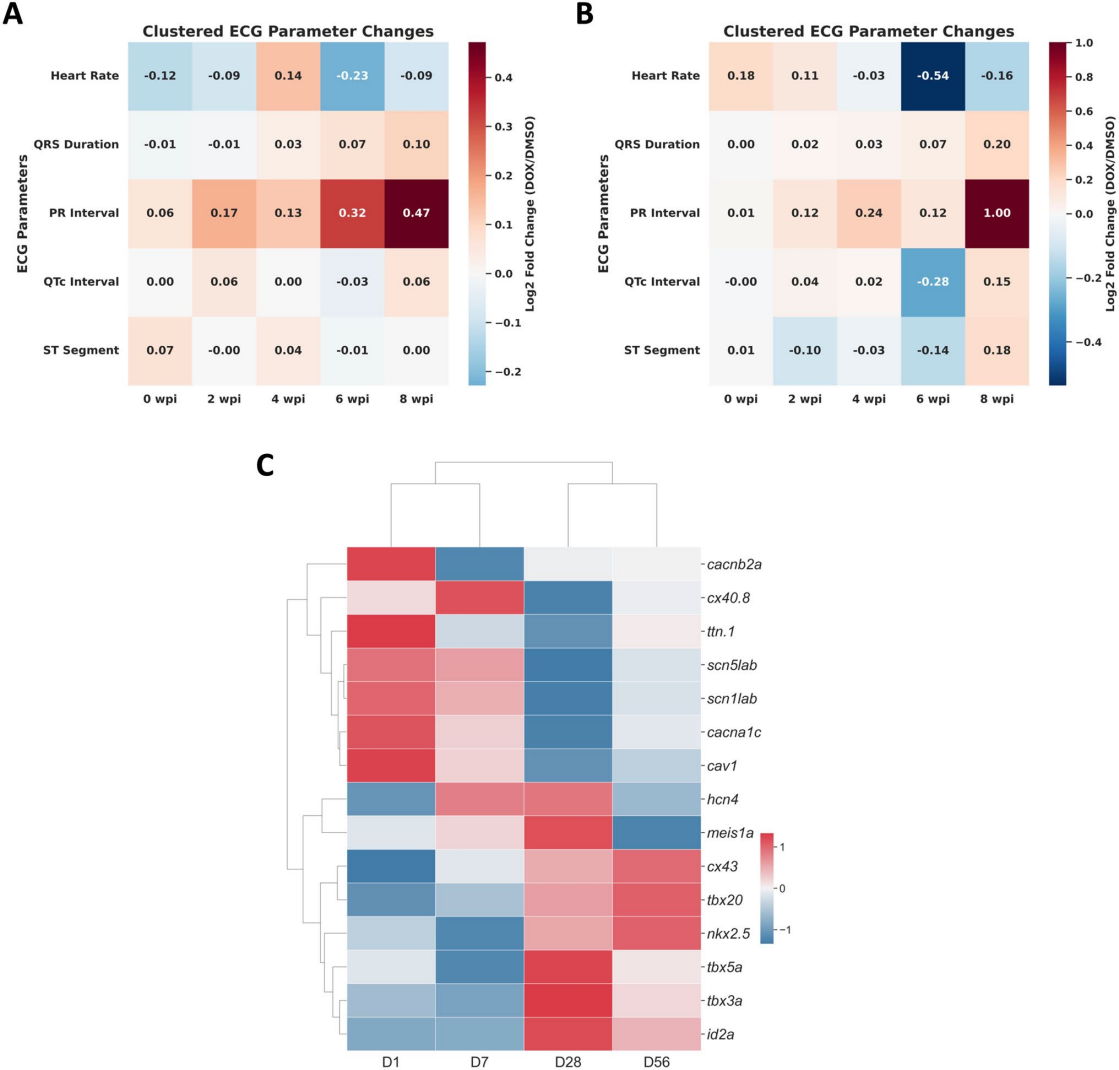
ECG alterations and associated transcriptomic signatures in DIC. Heatmaps showed log2 FC of ECG parameters relative to 2% DMSO controls after 20 mg/kg (**A**) and 40 mg/kg (**B**) DOX exposure. Both doses were associated with progressive PR interval prolongation, with more pronounced effects at 40 mg/kg. Higher-dose DOX also caused marked bradycardia, whereas changes in QRS duration, QTc interval, and ST segment were modest. **C** Heatmap of a curated, literature-informed set of PR interval/AV conduction-related genes at 1, 7, 28, and 56 days post DOX treatment. Gene expression values were normalized using Z-score transformation. Key conduction genes (*scn5lab*, *scn1lab*), gap junction proteins (*cx43*, *cx40.8*), and transcription factors (*nkx2.5*, *tbx* family) were dysregulated, observed alongside PR interval changes in this model

Given the prominent ECG alterations observed in our electrophysiological assessments, we generated a clustered heatmap with annotated alterations in ECG parameters to assess dose-dependent electrophysiological effects of DOX. We compared Log2 FC in ECG parameters relative to DMSO after treatment with 20 mg/kg and 40 mg/kg DOX. At 20 mg/kg DOX (Fig. 6A), PR interval exhibited the most consistent and progressive increased across time points, with Log2 FC rising from 0.06 at baseline to 0.47 at 8 wpi. This indicated a dose-dependent conduction delay. In contrast, QRS duration and QTc interval showed only mild fluctuations without sustained alterations, and ST segment changes remained minimal. Heart rate displayed variable responses, with a transient increased at 4 wpi (0.14) followed by a reduction at 6 wpi (− 0.23). At 40 mg/kg DOX (Fig. 6B), PR interval prolongation was more pronounced, peaking at a Log2 FC of 1.00 at 8 wpi. Heart rate demonstrated a marked decline at 6 wpi (− 0.54), indicating dose-related bradycardia. QRS duration and QTc interval showed modest increases at later stages, whereas ST segment changes were again variable and less prominent. The consistent clustering pattern across the two independent heatmaps highlights the reproducibility of these findings. Collectively, these results indicate that DOX administration preferentially disrupts AV conduction (PR interval) and autonomic heart rate regulation, while exerting comparatively minor effects on ventricular depolarization, repolarization, and ST segment morphology.

### Transcriptomic Alterations in PR Interval-Associated Genes Associated with DOX-Induced Conduction Abnormalities

To further delineate the molecular mechanisms accompanying the observed electrophysiological and structural alterations, we conducted RNA sequencing (RNA-seq) profiling at 1, 7, 28, and 56 days post DOX administration in zebrafish. These timepoints were selected to capture early-to-late transcriptional responses during the period in which cardiac injury biomarkers and ECG abnormalities became evident. We examined the expression patterns of genes previously implicated in AV conduction and PR interval regulation [32]. Analysis of PR interval-associated genes revealed significant dysregulation of key cardiac electrophysiological components, including voltage-gated sodium channels (*scn5lab*, *scn1lab*), l-type calcium channels (*cacna1c*, *cacnb2a*), transcription factors (*tbx3a*, *tbx5a*, *tbx20*, *nkx2.5*, *meis1a*, *id2a*), gap junction proteins (*cx43*, *cx40.8*) essential for intercellular conduction and other related genes (*hcn4*, *cav1*, *ttn.1*) (Fig. 6C).

Among these genes, *scn1lab* (encoding a sodium channel subunit) showed progressive downregulation over time, consistent with reduced excitability and slowed impulse propagation in models of conduction remodeling. In contrast, *cx43*, a critical component of gap junctions, showed sustained upregulation over time, suggesting transcriptional remodeling of intercellular coupling in response to DOX exposure. Meanwhile, *nkx2.5*, a master regulator of cardiac development and structural integrity, was progressively upregulated at later stages (28–56 dpi), compatible with an adaptive stress-responsive program during ongoing injury. Together, these temporal patterns indicate that conduction-related (*scn1lab*), coupling-related (*cx43*), and transcriptional regulatory (*nkx2.5*) programs are differentially modulated over the course of DIC.

Overall, the results identify candidate molecular pathways that co-occur with the progression of ECG abnormalities in the zebrafish model. In particular, the temporal expression dynamics of *scn1lab*, *cx43*, and *nkx2.5* highlight transcriptional changes associated with the observed ECG abnormalities following DOX treatment, particularly PR interval prolongation. These findings demonstrate a temporal association between transcriptional changes in conduction-related genes and the observed electrophysiological abnormalities following DOX exposure.

## Discussion

This study established adult zebrafish as a robust and clinically relevant model for investigating DIC using an integrated approach to combine real-time electrophysiological monitoring with temporal molecular profiling. Our findings demonstrated that zebrafish could faithfully recapitulate key pathophysiological features of human DIC, including structural alterations, elevated cardiac biomarkers, and ECG abnormalities that parallel clinical observations. The development of dilated cardiomyopathy, ventricular enlargement, and increased cardiac troponin I levels in DOX-treated zebrafish could closely mirror the clinical manifestations observed in cancer patients receiving anthracycline therapy, thereby validating the translational relevance of this model.

Critically, our zebrafish model exhibited electrophysiological responses that closely resemble human cardiac dysfunction patterns. The dose-dependent prolongation of PR interval and QRS duration, along with P wave widening, could mirror the ECG abnormalities reported in clinical studies of both pediatric and adult cancer patients treated with anthracyclines. This electrophysiological resemblance, together with the genetic homology between zebrafish and humans, establishes this model as an invaluable platform for mechanistic studies and therapeutic development in cardiovascular toxicology.

Histological and morphometric analyses confirmed that DOX exposure in adult zebrafish induces dilated cardiomyopathy-like changes, including ventricular enlargement and cardiomyocyte rarefaction (Figs. 1 and 2), consistent with previous reports in both zebrafish and mammalian models [47, 48]. The detection of myocardial vacuolization as early as 2 wpi underscores the rapid onset of subcellular injury. These changes were paralleled by elevated circulating cardiac troponin I, a highly specific biomarker for myocardial damage in clinical settings [43]. Together, these findings support the pathological relevance of zebrafish for modeling DOX-induced myocardial injury.

Among ECG indices, PR interval prolongation emerged as the most consistent and dose-dependent abnormality (Fig. 6A, B). While QRS and QTc changes were modest, PR interval prolongation became apparent from mid to late time points and was more pronounced at higher DOX doses. This pattern aligns with clinical reports and reviews showing that anthracycline therapy can produce AV conduction disturbances (including isolated reports of PR interval prolongation and AV block) in some patients [49]. Moreover, our clustered heatmap analysis demonstrated reproducible dose-dependent effects, supporting the PR interval as a sensitive readout of conduction perturbation in the zebrafish model. Notably, PR interval changes were detectable before overt morphological failure in our dataset, suggesting potential utility for earlier surveillance of cardiotoxicity.

Clinical translation of PR interval prolongation warrants cautious interpretation. In anthracycline-treated patients, ECG abnormalities, including PR prolongation and AV block, have been reported, but their frequency and pattern vary across cohorts, cumulative dose, co-therapies, and surveillance timing. Human biomarkers such as troponins and natriuretic peptides support early myocardial injury during therapy, yet they are not specific to conduction-system remodeling. Moreover, human cardiac transcriptomic data collected during anthracycline exposure remain limited; therefore, the molecular signatures identified here should be viewed as hypothesis-generating correlates that require cross-species validation and functional studies.

Transcriptomic profiling further identified dysregulation of sodium channel subunits (*scn5lab*, *scn1lab*), gap junction proteins (*cx43*, *cx40.8*), and cardiac transcription factors (*nkx2.5*, *tbx* family), all of which have been implicated in cardiac conduction biology (Fig. 6C). Specifically, the progressive downregulation of *scn1lab*, which may be associated with impaired depolarization and slowed conduction. Conversely, the upregulation of *cx43* might represent a compensatory mechanism aimed at preserving intercellular coupling under electrophysiological stress. Similarly, the sustained increase in *nkx2.5*, a key cardiac transcription factor [50], is also compatible with an adaptive transcriptional response during ongoing injury. Importantly, because these molecular readouts were derived from RNA-seq without direct functional perturbation, the present study does not establish a direct mechanistic linkage between specific pathways and PR interval prolongation. Instead, the data support a correlative association between conduction-related transcriptomic remodeling and electrophysiological alterations, which may be mediated by intermediate processes such as cardiomyocyte injury, structural remodeling, fibrosis-like changes, or autonomic modulation, which were not directly quantified here. Future studies integrating targeted genetic or pharmacologic manipulation with longitudinal electrophysiological measurements will be necessary to determine causality and clarify which molecular changes are drivers versus downstream responses.

The zebrafish model offers several distinct advantages for DIC research that address current limitations in the field. The ability to perform high-throughput screening with real-time physiological monitoring enables rapid evaluation of cardioprotective interventions that would be prohibitively expensive and time-consuming in mammalian models [51]. The genetic tractability of zebrafish allows for targeted manipulation of specific pathways identified in our transcriptomic analysis, facilitating mechanistic validation and therapeutic target development. Additionally, the optical transparency of zebrafish larvae and the availability of transgenic lines with fluorescent cardiac markers enable non-invasive, longitudinal assessment of cardiac function and cellular responses [52].

Several limitations should be acknowledged. First, although the integrated electrophysiological and transcriptomic analyses provide insight into DOX-induced cardiac alterations, the transcriptomic findings are observational and were not independently validated by orthogonal assays (e.g., qPCR, in situ hybridization/WISH, or protein-level methods such as IHC). Accordingly, in the absence of functional perturbation experiments, the observed transcriptional changes, including those in conduction-related genes, should be interpreted as correlative with PR interval prolongation and other ECG phenotypes rather than definitive causal drivers; moreover, direct human transcriptomic evidence linking conduction-pathway remodeling to PR changes during anthracycline therapy remains limited, and our molecular results should therefore be viewed as hypothesis-generating. In this study, the single-dose DOX model in zebrafish with longitudinal follow-up best represents the acute-to-subacute injury phase and subsequent remodeling features within the experimental timeframe, rather than fully replicating the cumulative-dose, long-term chronic anthracycline cardiomyopathy observed in patients. Second, while both male and female zebrafish were randomly included, the study was not powered for sex-stratified analyses, which may limit direct translational extrapolation given known sex-dependent susceptibility to anthracycline cardiotoxicity. Third, although appropriate vehicle controls were included, the relatively high concentration of DMSO used as a solvent may have influenced baseline physiological states. This potential confounding factor should be considered when interpreting subtle molecular and electrophysiological changes. Finally, ECG recordings were obtained under anesthesia, which can influence heart rate and conduction parameters; although all groups were assessed under identical conditions, residual effects of anesthetic depth and inter-individual variability cannot be fully excluded. Despite these limitations, the present study establishes a framework for integrating ECG-based phenotyping with molecular profiling and provides a foundation for future mechanistic and translational investigations.

Our integrated approach establishes a foundation for several important research directions. The identified cardiac electrophysiological components warrant further investigation through genetic manipulation studies to establish causative relationships and evaluate therapeutic potential. The development of zebrafish-based screening platforms for cardioprotective compounds could accelerate the identification of clinically viable interventions. Additionally, the temporal molecular signatures identified in our study could be validated as predictive biomarkers in clinical cohorts to enable early detection and risk stratification.

The mechanistic insights provided by our study also suggest novel therapeutic strategies beyond traditional antioxidant approaches. Targeting the cardiac electrophysiological signaling pathway might provide complementary cardioprotective effects. Furthermore, our demonstration of the importance of dose intensity optimization provides a framework for developing personalized dosing algorithms that balance anti-tumor efficacy with cardiotoxic risk based on individual patient characteristics and molecular profiles.

In conclusion, this study advances our understanding of DIC by establishing a clinically relevant zebrafish model that enables integrated investigation of the structural, functional, and molecular aspects of anthracycline cardiotoxicity. The molecular mechanisms identified provide novel therapeutic targets and early biomarkers, while the demonstrated importance of dose intensity optimization offers immediate clinical applications. These findings collectively contribute to the development of more effective strategies to prevent and manage DIC, ultimately improving outcomes for cancer patients receiving life-saving anthracycline therapy.

## Data Availability

The data that support the findings of this study are not openly available due to reasons of sensitivity and are available from the corresponding author upon reasonable request.

## Abbreviations

AV: Atrioventricular
DOX: Doxorubicin
DIC: DOX-induced cardiotoxicity
DMSO: Dimethyl sulfoxide
Dpi: Days post injection
ECG: Electrocardiography
H&E staining: Hematoxylin and Eosin staining
IP: Intraperitoneal
Log2 FC: Log2 fold change
LVEF: Left ventricular ejection fraction
Ms: Millisecond
PBS: Phosphate-buffered saline
QTc interval: Corrected QT interval
RLE: Relative log expression
RNA-seq: RNA sequencing
RIN: RNA integrity number
SD: Standard deviation
Wpi: Weeks post injection
